# Correction: Dementias platform UK clinical studies and great minds register: protocol of a targeted brain health studies recontact database

**DOI:** 10.1136/bmjopen-2020-040766corr1

**Published:** 2020-12-23

Koychev I, Young S, Holve H, *et al*. Dementias Platform UK Clinical Studies and Great Minds Register: protocol of a targeted brain health studies recontact database. *BMJ Open* 2020;10:e040766. doi: 10.1136/bmjopen-2020-040766

This article was previously published with an error. Authors missed to include the below acknowledgement section.

The authors acknowledge the technical support by the Swansea University UKSeRP team (www.ukserp.ac.uk) in the implementation of the protocol into research infrastructure: Chris Orton provided organisational oversight, Justin Biddle designed and implemented the Great Minds data collection website according to specification, Laura North developed the participant selection and feasibility tools for Great Minds and the Clinical Studies Register according to specification. We also acknowledge the contribution of the staff of DPUK cohorts which have so far adopted the Great Minds and Clinical Studies Register re-contact platform: Airwave Health Monitoring Study (Paul Elliott, Andy Heard and Gao He, Imperial College London), HealthWise Wales (Shantini Paranjothy and Charlotte Bonner-Evans, Cardiff University), Brains for Dementia Research (Alan Thomas and Nicky Barnett, Newcastle University), England sites of European Prevention of Alzheimer’s Disease platform (Craig Ritchie, University of Edinburgh and Vanessa Raymont, University of Oxford) and Sleep Quest study (Elizabeth Coulthard, Jonathan Blackman, Neil Carrigan and Alfie Wearn, Bristol University). The project was completed through Medical Research Council funding (MR/L023784/2).

